# Treatment Failure and Long-Term Prescription Risk for Guideline-Recommended Hypnotics in Japan

**DOI:** 10.1001/jamanetworkopen.2024.6865

**Published:** 2024-04-17

**Authors:** Masahiro Takeshima, Kazuhisa Yoshizawa, Masaya Ogasawara, Mizuki Kudo, Yu Itoh, Naoko Ayabe, Kazuo Mishima

**Affiliations:** 1 Department of Neuropsychiatry, Akita University Graduate School of Medicine, Akita, Japan; 2 Department of Regional Studies and Humanities, Faculty of Education and Human Studies, Akita University, Akita, Japan

## Abstract

**Importance:**

Although insomnia guidelines recommend the use of several individual hypnotics, the most useful hypnotic for treating insomnia in a clinical setting remains unclear.

**Objective:**

To determine which guideline-recommended hypnotics have lower risks of monotherapy failure and which hypnotics have a higher risk of long-term prescription for insomnia treatment.

**Design, Setting, and Participants:**

This retrospective observational cohort study used data from the Japan Medical Data Center Claims Database from April 1, 2005, to March 31, 2021. Participants included adults whose first prescribed pharmaceutical treatment for insomnia was guideline-recommended hypnotic monotherapy. Data were analyzed from December 24, 2022, to September 26, 2023.

**Exposures:**

Suvorexant, ramelteon, eszopiclone, zolpidem, and triazolam monotherapy.

**Main Outcomes and Measures:**

The primary outcome was monotherapy failure, defined as a change in hypnotic or having an additional hypnotic prescribed for insomnia within 6 months of the first prescription of a guideline-recommended hypnotic monotherapy. The secondary outcome was monotherapy discontinuation, defined as no prescription of any hypnotic for 2 consecutive months within 6 months after prescribing a guideline-recommended hypnotic in patients for whom monotherapy did not fail. Monotherapy failure and discontinuation were compared using Cox proportional hazards and logistic regression models, respectively.

**Results:**

The study included 239 568 adults (median age, 45 [IQR, 34-55] years; 50.2% women) whose first prescription for insomnia was guideline-recommended hypnotic monotherapy. During the 6-month follow-up period, 24 778 patients (10.3%) experienced failure of monotherapy with a guideline-recommended hypnotic. In comparison with eszopiclone, there were more cases of monotherapy failure for ramelteon (adjusted hazard ratio [AHR], 1.23 [95% CI], 1.17-1.30; *P* < .001), fewer cases for zolpidem (AHR, 0.84 [95% CI, 0.81-0.87]; *P* < .001) and triazolam (AHR, 0.82 [95% CI, 0.78-0.87]; *P* < .001), and no significant difference between suvorexant and eszopiclone. Among those without monotherapy failure, monotherapy was discontinued in 84.6% of patients, with more discontinuations for ramelteon (adjusted odds ratio [AOR], 1.31 [95% CI, 1.24-1.40]; *P* < .001) and suvorexant (AOR, 1.20 [95% CI, 1.15-1.26]; *P* < .001) than for eszopiclone and no significant difference between zolpidem or triazolam and eszopiclone.

**Conclusions and Relevance:**

Due to uncontrolled confounding factors in this cohort study, no conclusions regarding the pharmacologic properties of guideline-recommended hypnotics can be drawn based on these results. Further studies accounting for confounding factors, including diagnoses of chronic vs acute insomnia disorder, insomnia and psychiatric symptom severity, and physician attitudes toward hypnotic prescription, are needed.

## Key Points

**Question:**

Which guideline-recommended hypnotics have lower risks of monotherapy failure in treating insomnia in a clinical setting?

**Findings:**

In this cohort study of 239 568 patients whose initial pharmacotherapy for insomnia was monotherapy with a guideline-recommended hypnotic, 10% had their hypnotic changed or had another hypnotic added within 6 months. Zolpidem and triazolam were associated with fewer monotherapy failures than eszopiclone, whereas ramelteon was associated with more; eszopiclone and suvorexant were comparable.

**Meaning:**

Because of the inconsistency between randomized clinical trial results and clinical setting results, randomized clinical trials directly comparing guideline-recommended hypnotics are needed.

## Teaser

This cohort study examines the risk of monotherapy failure and risk of long-term prescription for guideline-recommended hypnotics prescribed for insomnia disorders among adults in Japan.

## Introduction

Chronic insomnia is one of the most common sleep disorders worldwide, with an estimated prevalence of 6% to 10%.^1^ It is characterized by difficulty initiating or maintaining sleep despite adequate sleep opportunity, resulting in daytime impairment of and interference with social, occupational, and other functions.^2^ Insomnia not only increases the risk of mental and physical illness^3^ but also results in increased economic costs,^4,5^ decreased work productivity,^6,7,8^ and increased workplace^9^ and traffic accidents.^10^

The main treatments for chronic insomnia disorders involve psychotherapy and pharmacotherapy. Most clinical guidelines recommend cognitive-behavioral therapy for insomnia (CBT-I) as a first-line treatment because of its superior benefit-to-harm ratio compared with that of pharmacotherapy.^11,12,13^ Reportedly, the acceptability of CBT-I and pharmacotherapy do not differ, and CBT-I results in higher sleep efficiency after treatment and in the 1- to 3-month and 4- to 11-month follow-up periods.^14^ However, because CBT-I is not available for all patients with chronic insomnia disorders, many affected patients are treated with pharmacotherapy.^15,16^

Some guidelines recommend individual hypnotics for insomnia disorders.^11,12,16,17^ The 2016 American College of Physicians guidelines recommend eszopiclone, zolpidem, and suvorexant for insomnia disorder in adults.^11^ The 2017 American Academy of Sleep Medicine Clinical Practice Guideline recommends triazolam, ramelteon, and zaleplon for sleep-onset insomnia; suvorexant and temazepam for sleep-maintenance insomnia; and zolpidem and eszopiclone for both sleep-onset and sleep-maintenance insomnia.^16^ Knowledge regarding guideline-recommended hypnotics that are most useful for treating insomnia disorders would be beneficial for physicians, patients, and caregivers, but the existing guidelines do not indicate this.^11,12,13,16^ To date, no randomized clinical trials (RCTs) have compared individual guideline-recommended hypnotics within a single trial. Recently, a network meta-analysis of placebo-controlled, double-blind trials^18^ compared different pharmacologic treatments for insomnia and concluded that eszopiclone had one of the best profiles among them. However, these results should be interpreted cautiously because the study included few RCTs that directly compared individual hypnotics; therefore, most differences in efficacy, tolerability, and safety between individual hypnotics were derived from indirect comparisons.^18^ One solution to this is to examine the results of the literature obtained from the network meta-analysis using data in a clinical setting adjusted for possible confounders. Additionally, when data from clinical trials do not match patterns in clinical settings, evaluating the potential reasons is important.

Long-term prescription of hypnotics is another important issue associated with pharmacotherapy for insomnia disorders. Because the long-term use of benzodiazepine receptor agonists (BzRAs), the most commonly prescribed class of hypnotics, can have harmful effects,^19^ most guidelines only recommend their short-term use for insomnia.^13,16,20^ However, despite warnings regarding the risks associated with long-term BzRA use and corresponding policy interventions in several countries,^21,22,23,24,25^ long-term use of hypnotics continues worldwide.^26,27,28^ A cohort study conducted using a Finnish national database to examine factors associated with long-term BzRA use^29^ reported that zolpidem had a lower risk of long-term prescription compared with diazepam. However, the participants were not patients with insomnia, and the study did not investigate the risks of long-term prescription of eszopiclone or triazolam, which are recommended for insomnia by several guidelines.^29^ In addition, previous studies may not have accurately assessed the risks of long-term use of individual hypnotics because they did not consider switching to or adding other hypnotics.^27,29^ For example, if a hypnotic is ineffective, the duration of the prescription will be shorter, and it will be longer if an additional hypnotic is added; thus, the duration of the prescription is influenced by the prescribing behavior of the physician and the treatment preferences of the patient. Therefore, which guideline-recommended hypnotics are more likely to be prescribed for the long term remains unclear. To determine the risk of long-term prescription of individual guideline-recommended hypnotics, it is necessary to evaluate not only the duration of prescription but also account for switching and concomitant use of hypnotics.

To examine guideline-recommended hypnotics that are most useful in treating insomnia disorders and those associated with risks of long-term prescription, we conducted a retrospective cohort study using a large-scale Japanese claims database. We hypothesized that (1) eszopiclone would be the least likely to fail in the treatment of insomnia among guideline-recommended hypnotics, as in the previous study,^18^ and (2) the novel hypnotics suvorexant and ramelteon would have a lower risk of long-term prescription because they are not addictive.

## Methods

This cohort study was approved by the Ethics Committee of the Akita University Graduate School of Medicine and the Faculty of Medicine Ethical Committee for Human Research and was conducted in accordance with the Declaration of Helsinki.^30^ Informed consent was not required owing to the use of deidentified retrospective data. This study was reported in accordance with the Strengthening the Reporting of Observational Studies in Epidemiology (STROBE) reporting guideline.

### Study Design and Data Source

This retrospective cohort study used data from the Japan Medical Data Center (JMDC) Claims Database, the largest health insurance claims database in Japan.^31^ This database contains deidentified claims data for health insurance provider company employees and their family members younger than 75 years as part of the Japanese union-managed health insurance system. This includes demographic and medical data (eg, information regarding diagnoses, prescriptions, procedures, medical services, costs, etc) based on the claims received by insured individuals (outpatient, inpatient, and pharmacy) from medical institutions. An identifier is assigned to each individual to link their claims records across medical facilities, making it possible to track their movement and treatment across medical facilities as long as they are covered by health insurance societies affiliated with the JMDC database. The JMDC database contained claims data dated between April 1, 2005, and March 31, 2021, for 11 244 687 insured individuals. In this study, the following information was extracted for each month during this period: age, sex, names of hypnotics prescribed based on the Anatomical Therapeutic Chemical code, prescription instructions for hypnotics (regular, as needed, or both), names of concomitant psychotropic drugs prescribed (based on the Anatomical Therapeutic Chemical code), and comorbid physical and psychiatric disorders based on the *International Statistical Classification of Diseases, Tenth Revision*. Details regarding the information that was extracted are provided in the eMethods and eTable 1 in Supplement 1.

### Study Population and Cohort Selection

Adult patients 20 years or older who were prescribed certain guideline-recommended hypnotics (triazolam, zolpidem, eszopiclone, ramelteon, and suvorexant) for the first time after joining a health insurance society affiliated with and included in the JMDC database were considered eligible to participate.^11,12,13,16^ Some guideline-recommended hypnotics were not included in this study: temazepam, zaleplon, and doxepin are not approved for use in Japan, and melatonin is indicated for difficulty falling asleep associated with childhood neurodevelopmental disorders but not for insomnia disorders. Trazodone is indicated for depression and depressive states but not insomnia disorders and was not included in the main analysis.

Patients who were prescribed 2 or more hypnotics in the first month of their first prescription were excluded because it was not possible to determine whether the hypnotics were prescribed at the same time or switched within the month or whether an additional hypnotic was added within the month. Additionally, patients diagnosed with sleep-disordered breathing, central hypersomnia, or circadian rhythm sleep-wake disorder in the month of their first prescription were excluded. Patients who were prescribed hypnotics within 6 months of joining a health insurance society affiliated with and included in the JMDC database were also excluded, as they may previously have been prescribed hypnotics covered by the National Health Insurance or a health insurance not included in the JMDC database. Moreover, eligible patients were followed up monthly for 6 months from the month in which hypnotics were first prescribed. If monotherapy was stopped after the fifth consecutive month, then that hypnotic prescription had to be followed up for 7 months to determine the primary end point of monotherapy failure and the secondary end point of monotherapy discontinuation. Therefore, patients who were first prescribed hypnotics after October 1, 2020, were excluded. Eligible patients were followed up monthly for 6 months from their first prescription of any hypnotic that can be prescribed in Japan (eTables 1-4 in Supplement 1).

### Definition of Events

If a guideline-recommended hypnotic was switched to another hypnotic (guideline-recommended or not) or if another hypnotic (guideline-recommended or not) was additionally prescribed before the first guideline-recommended hypnotic was no longer prescribed for 2 consecutive months after the initial prescription, the guideline-recommended hypnotic monotherapy was considered to have failed. If a guideline-recommended hypnotic was not prescribed for 2 consecutive months without being switched to another hypnotic or another hypnotic being additionally prescribed, the guideline-recommended hypnotic was considered to have been discontinued. If a patient who received their first prescription of hypnotic monotherapy for 5 consecutive months was not prescribed a hypnotic in the sixth month, the data for the seventh month were also examined. If no hypnotic was prescribed in the seventh month, the patient was considered to have discontinued the hypnotic; if a hypnotic was prescribed in the seventh month, the patient was considered to have not discontinued hypnotic therapy.

Patients who withdrew from the JMDC database prior to a monotherapy failure or monotherapy discontinuation event were considered censored. Specific examples of monotherapy failure or monotherapy discontinuation events are shown in eFigure 1 in Supplement 1.

### Primary and Secondary Outcomes

The primary objective was to examine which guideline-recommended hypnotics have lower risks of monotherapy failure in treating insomnia. We considered monotherapy failure within 6 months of initial prescription as a surrogate measure and selected it as the primary end point.

The secondary objective was to determine which guideline-recommended hypnotics were associated with a higher risk of long-term prescription considering switching to other hypnotics or adding other hypnotics. In clinical practice, hypnotics are often changed when one is ineffective. If a hypnotic causes intolerable adverse effects, the drug is often discontinued and switched to another hypnotic, either immediately or after a period of time. We defined monotherapy discontinuation within 6 months of initial prescription as the secondary end point for patients who did not experience failure of monotherapy with a guideline-recommended hypnotic.

### Statistical Analysis

Data were analyzed from December 24, 2022, to September 26, 2023. Nonnormally distributed continuous and categorical variables are expressed as medians and IQRs or as numbers and percentages, respectively. Cox proportional hazards regression modeling was used to determine which guideline-recommended hypnotics had a higher risk of monotherapy failure, adjusting for age group (20-39, 40-64, and ≥65 years), sex, administration of the first prescribed hypnotic (regular prescription, prescription as needed, or both), concomitant anxiolytics, concomitant antidepressants, concomitant antipsychotics, comorbid psychiatric disorders, and comorbid physical disorders in the month of the first prescription. These covariates were selected based on previous studies examining the risk factors for long-term prescribing of BzRAs.^29^ Details regarding testing the Cox proportional hazards regression model assumptions using Stata, version 13.0 (StataCorp LLC), are provided in the eMethods and eFigure 2 in Supplement 1. To confirm robustness, a sensitivity analysis was performed by excluding patients 65 years or older due to insufficient evidence on the efficacy and safety of hypnotics in elderly patients with insomnia.^16^ In addition, because patients with insomnia and comorbid psychiatric disorders are less likely to respond to hypnotics compared with those without comorbid psychiatric disorders,^32^ a sensitivity analysis was performed excluding mood disorder, anxiety disorder, mood or anxiety disorder, and any psychiatric disorders. Prescribing behavior for hypnotics may have changed over time because suvorexant, eszopiclone, and ramelteon were introduced during the study period. Therefore, another sensitivity analysis was performed excluding patients who were first prescribed hypnotics before November 2014, when suvorexant, the newest of the guideline-recommended hypnotic, was introduced. In clinical settings, clinicians often use medications that can be helpful for both major depressive disorder and insomnia symptoms to prevent polypharmacy. Thus, patients who were not prescribed trazodone to be taken before bedtime in the first month of their first prescription of a guideline-recommended hypnotic were evaluated for monotherapy failure, considering the use of trazodone prescribed to be taken before bedtime as well as all hypnotics. Binary logistic regression analysis was used to examine the associations between guideline-recommended hypnotics and monotherapy discontinuation after adjusting for the same covariates used in the Cox proportional hazards regression model. All statistical analyses, other than Cox proportional hazard regression model assumption testing, were performed using SPSS Statistics, version 28.0 (IBM Corp). Two-sided *P* < .05 was considered significant.

## Results

This study included 239 568 adult patients with insomnia who were prescribed guideline-recommended hypnotic monotherapy in the month of the first hypnotic prescription. The participant selection flowchart is shown in eFigure 3 in Supplement 1. The clinical and demographic characteristics of the participants in the month of the first hypnotic prescription are summarized in Table 1. The median age was 45 (IQR, 34-55) years; 50.2% of patients were women and 49.8% were men. Zolpidem was the first prescribed hypnotic for 56.6% of patients; suvorexant, 15.7%; eszopiclone, 14.2%; triazolam, 7.5%; and ramelteon, 6.1%. The zolpidem group had the lowest percentage of patients with mood disorders (20.7%), followed by the triazolam (26.3%), ramelteon (32.1%), suvorexant (33.8%), and eszopiclone (34.7%) groups. A similar trend was observed for neurotic, stress-related, and somatoform disorders, with the zolpidem group having the lowest percentage of anxiety disorder (22.8%), followed by the triazolam (23.3%), ramelteon (31.2%), eszopiclone (3.17%), and suvorexant 31.8%) groups.

**Table 1.** Clinical and Demographic Characteristics of Included Patients in the Month of First Prescription of Hypnotics

Characteristic	Treatment group, No. (%) of patients^a^					
	Eszopiclone (n = 34 050)	Ramelteon (n = 14 523)	Suvorexant (n = 37 547)	Zolpidem (n = 135 492)	Triazolam (n = 17 956)	All (N = 239 568)
Age group, y						
20-39	12 869 (37.8)	6223 (42.8)	13 208 (35.2)	47 510 (35.1)	6823 (38.0)	86 633 (36.2)
40-64	18 788 (55.2)	7263 (50.0)	21 454 (57.1)	78 204 (57.7)	9849 (54.9)	135 558 (56.6)
≥65	2393 (7.0)	1037 (7.1)	2885 (7.7)	9778 (7.2)	1284 (7.2)	17 377 (7.3)
Sex						
Men	17 885 (52.5)	7671 (52.8)	20 799 (55.4)	64 292 (47.5)	8703 (48.5)	119 350 (49.8)
Women	16 165 (47.5)	6852 (47.2)	16 748 (44.6)	71 200 (52.5)	9253 (51.5)	120 218 (50.2)
Prescription instruction						
As needed	12 273 (36.0)	1891 (13.0)	8382 (22.3)	46 644 (34.4)	4628 (25.8)	73 818 (30.8)
Regular	21 777 (64.0)	12 632 (87.0)	29 165 (77.7)	88 848 (65.6)	13 328 (74.2)	165 750 (69.2)
Physical comorbidities						
Hypertension	5747 (16.9)	2554 (17.6)	7554 (20.1)	24 233 (17.9)	2975 (16.6)	43 063 (18.0)
Hyperlipidemia	5036 (14.8)	2434 (16.8)	6701 (17.8)	21 406 (15.8)	2798 (15.6)	38 375 (16.0)
Diabetes	3189 (9.4)	1464 (10.1)	4118 (11.0)	13 196 (9.7)	1424 (7.9)	23 391 (9.8)
Cancer	2664 (7.8)	781 (5.4)	2120 (5.6)	12 094 (8.9)	736 (4.1)	18 395 (7.7)
Rheumatoid arthritis	413 (1.2)	134 (0.9)	346 (0.9)	1540 (1.1)	146 (0.8)	2579 (1.1)
Parkinson disease	208 (0.6)	149 (1.0)	224 (0.6)	425 (0.3)	125 (0.7)	1131 (0.5)
Multiple sclerosis	42 (0.1)	5 (0.03)	41 (0.1)	122 (0.1)	7 (0.04)	217 (0.1)
Asthma	1835 (5.4)	848 (5.8)	2152 (5.7)	8401 (6.2)	1020 (5.7)	14 256 (6.0)
COPD	205 (0.6)	83 (0.6)	258 (0.7)	813 (0.6)	79 (0.4)	1438 (0.6)
Coronary artery disease	1253 (3.7)	623 (4.3)	1571 (4.2)	4791 (3.5)	567 (3.2)	8805 (3.7)
Pulmonary embolism	46 (0.1)	23 (0.2)	73 (0.2)	190 (0.1)	15 (0.1)	347 (0.1)
Deep vein thrombosis	305 (0.9)	93 (0.6)	241 (0.6)	1078 (0.8)	93 (0.5)	1810 (0.8)
Hypothyroid	472 (1.4)	227 (1.6)	558 (1.5)	1737 (1.3)	178 (1.0)	3172 (1.3)
Subarachnoid hemorrhage	93 (0.3)	41 (0.3)	105 (0.3)	290 (0.2)	14 (0.1)	543 (0.2)
Intracerebral hemorrhage	136 (0.4)	78 (0.5)	176 (0.5)	407 (0.3)	29 (0.2)	826 (0.3)
Cerebral infarction	458 (1.3)	246 (1.7)	650 (1.7)	1728 (1.3)	154 (0.9)	3236 (1.4)
Heart failure	1216 (3.6)	574 (4.0)	1507 (4.0)	3950 (2.9)	322 (1.8)	7569 (3.2)
Inflammatory bowel disease	220 (0.6)	60 (0.4)	177 (0.5)	737 (0.5)	69 (0.4)	1263 (0.5)
Psychiatric comorbidities						
Organic mental disorder	194 (0.6)	188 (1.3)	299 (0.8)	379 (0.3)	26 (0.1)	1086 (0.5)
Substance use disorder	280 (0.8)	121 (0.8)	380 (1.0)	761 (0.6)	105 (0.6)	1647 (0.7)
Schizophrenia	1795 (5.3)	866 (6.0)	2000 (5.3)	2369 (1.7)	818 (4.6)	7848 (3.3)
Mood disorders	11 800 (34.7)	4659 (32.1)	12 683 (33.8)	28 001 (20.7)	4719 (26.3)	61 862 (25.8)
Anxiety disorder	10 786 (31.7)	4525 (31.2)	11 947 (31.8)	30 873 (22.8)	4191 (23.3)	62 322 (26.0)
Disorders of adult personality and behavior	503 (1.5)	296 (2.0)	604 (1.6)	2034 (1.5)	173 (1.0)	3610 (1.5)
Personality disorder	89 (0.3)	46 (0.3)	106 (0.3)	151 (0.1)	25 (0.1)	417 (0.2)
Intellectual disability	66 (0.2)	123 (0.8)	98 (0.3)	118 (0.1)	18 (0.1)	423 (0.2)
Psychological development disorder	295 (0.9)	405 (2.8)	371 (1.0)	478 (0.4)	73 (0.4)	1622 (0.7)
BEDCA and UMD	499 (1.5)	342 (2.4)	488 (1.3)	731 (0.5)	143 (0.8)	2203 (0.9)
Psychotropic medications						
Antidepressants	9469 (27.8)	3403 (23.4)	10 151 (27.0)	22 720 (16.8)	3855 (21.5)	49 598 (20.7)
Trazodone before bedtime	429 (1.3)	174 (1.2)	522 (1.4)	837 (0.6)	163 (0.9)	2125 (0.9)
Anxiolytics	7867 (23.1)	2790 (19.2)	8031 (21.4)	28 401 (21.0)	4900 (27.3)	51 989 (21.7)
Antipsychotics	2616 (7.7)	1159 (8.0)	2943 (7.8)	3791 (2.8)	946 (5.3)	11 455 (4.8)

Abbreviations: BEDCA, behavioral and emotional disorders with onset usually occurring in childhood and adolescence; COPD, chronic obstructive pulmonary disease; UMD, unspecified mental disorder.

^a^ Percentages have been rounded and may not total 100.

**Figure 1.**
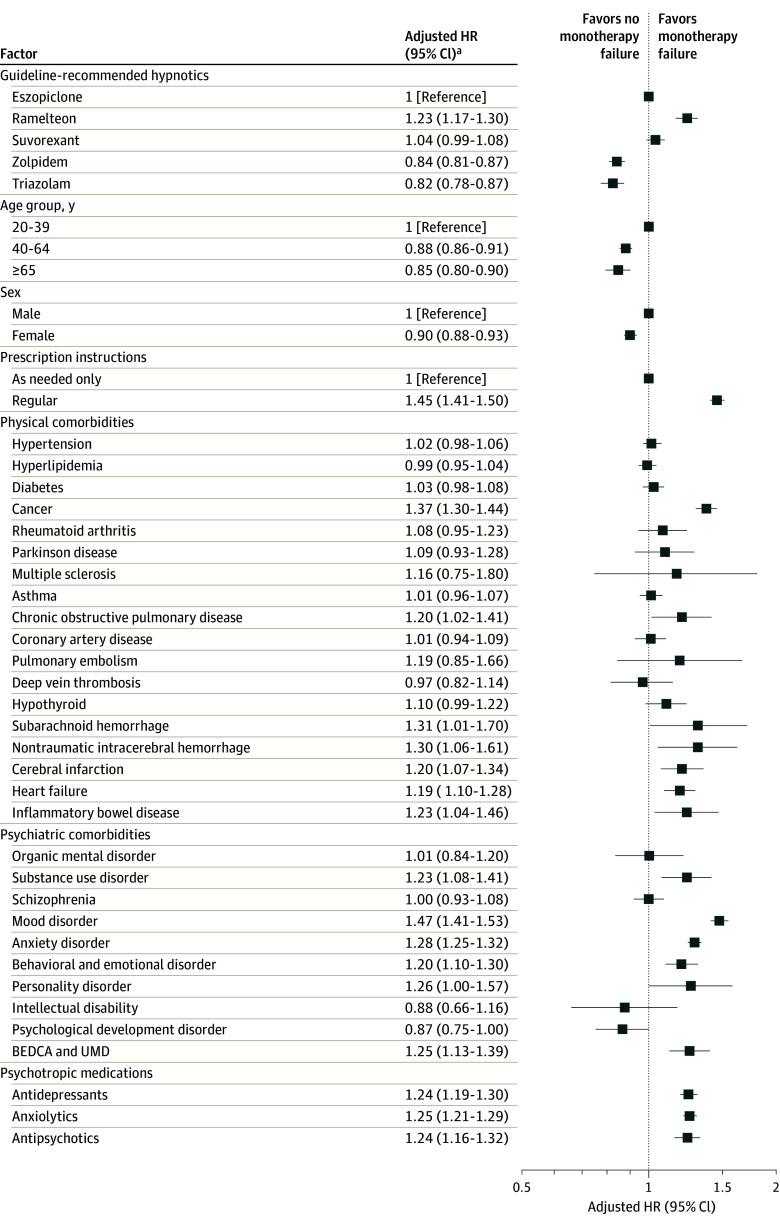
Factors Associated With Failure of Guideline-Recommended Hypnotic Monotherapy in Cox Proportional Hazards Regression Analysis Large hazard ratios (HRs) indicate more monotherapy failures, whereas small HRs indicate fewer monotherapy failures. BEDCA indicates behavioral and emotional disorders with onset usually occurring in childhood and adolescence; UMD, unspecified mental disorder. ^a^Adjusted for age group (20-39, 40-64, or ≥65 years), sex, administration instructions of the first prescribed hypnotic (regular prescription, prescription as needed, or both), concomitant anxiolytics, concomitant antidepressants, concomitant antipsychotics, comorbid psychiatric disorders, and comorbid physical disorders in the month of the first prescription.

Among patients with insomnia who started guideline-recommended hypnotic monotherapy, 10.3% experienced failure of monotherapy in less than 6 months (Table 2). Monotherapy failure was lowest for zolpidem (8.9%), followed by triazolam (9.4%), eszopiclone (11.9%), suvorexant (12.7%), and ramelteon (15.1%). Cox proportional hazards regression analysis showed that compared with eszopiclone, monotherapy failure was higher for ramelteon (adjusted hazard ratio [AHR], 1.23 [95% CI, 1.17-1.30]; *P* < .001) and lower for zolpidem (AHR, 0.84 [95% CI, 0.81-0.87]; *P* < .001) and triazolam (AHR, 0.82 [95% CI, 0.78-0.87]; *P* < .001). However, there was no significant difference in monotherapy failure between suvorexant and eszopiclone (AHR, 1.04 [95% CI, 0.99-1.08]; *P* = .09) (Figure 1 and eFigure 4 and eTable 5 in Supplement 1). Sensitivity analysis showed the same results except in the case of suvorexant, with fewer monotherapy failures with zolpidem and triazolam and more with ramelteon than with eszopiclone (eTable 6 in Supplement 1). Although eszopiclone and suvorexant did not differ in terms of monotherapy failure in the main analysis and sensitivity analyses excluding older patients, first prescriptions prior to the introduction of suvorexant, and mood disorder, suvorexant was associated with more monotherapy failures than eszopiclone in sensitivity analyses excluding anxiety disorder, mood or anxiety disorders, and any psychiatric disorder.

**Table 2.** Events Occurring Within 6 Months of First Prescription of Guideline-Recommended Hypnotics

Outcome	Treatment group, No. (%) of patients^a^					
	Eszopiclone (n = 34 050)	Ramelteon (n = 14 523)	Suvorexant (n = 37 547)	Zolpidem (n = 135 492)	Triazolam (n = 17 956)	All (N = 239 568)
Monotherapy failure	4062 (11.9)	2193 (15.1)	4767 (12.7)	12 066 (8.9)	1690 (9.4)	24 778 (10.3)
Monotherapy discontinuation	24 857 (73.0)	10 468 (72.1)	27 538 (73.3)	105 138 (77.6)	13 622 (75.9)	181 623 (75.8)
Continuation for 6 mo	3627 (10.7)	1268 (8.7)	3564 (9.5)	12 915 (9.5)	1897 (10.6)	23 271 (9.7)
Censored	1504 (4.4)	594 (4.1)	1678 (4.5)	5373 (4.0)	747 (4.2)	9896 (4.1)

^a^ Percentages have been rounded and may not total 100.

**Figure 2.**
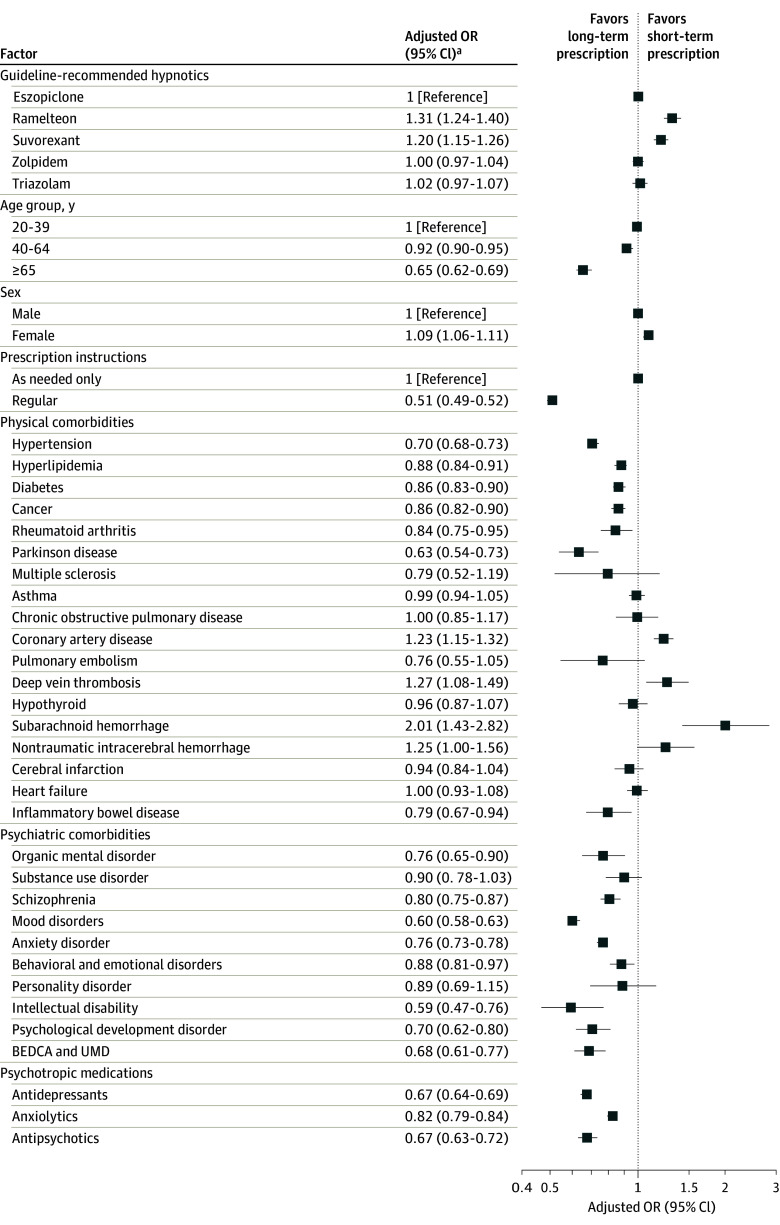
Factors Associated With Discontinuation of Guideline-Recommended Hypnotic Monotherapy in Logistic Regression Analysis Includes patients who did not experience failure of guideline-recommended monotherapy. Large odds ratios (ORs) indicate more monotherapy discontinuations, whereas small ORs indicate fewer monotherapy discontinuations. BEDCA indicates behavioral and emotional disorders with onset usually occurring in childhood and adolescence; UMD, unspecified mental disorder. ^a^Adjusted for age group (20-39, 40-64, or ≥65 years), sex, administration instructions of the first prescribed hypnotic (regular prescription, prescription as needed, or both), concomitant anxiolytics, concomitant antidepressants, concomitant antipsychotics, comorbid psychiatric disorders, and comorbid physical disorders in the month of the first prescription.

Among patients who did not experience failure of guideline-recommended hypnotic monotherapy, 84.6% discontinued hypnotics within 6 months (eTable 7 in Supplement 1). Monotherapy discontinuation was highest with zolpidem (85.2%), followed by ramelteon (84.9%), suvorexant (84.0%), triazolam (83.7%), and eszopiclone (82.9%). Monotherapy discontinuation was noted more in the ramelteon (adjusted odds ratio [AOR], 1.31 [95% CI, 1.24-1.40]; *P* < .001) and suvorexant (AOR, 1.20 [95% CI, 1.15-1.26]; *P* < .001) groups than in the eszopiclone group, but there was no significant difference between the zolpidem (AOR, 1.00 [95% CI, 0.97-1.04]; *P* = .97) or triazolam (AOR, 1.02 [95% CI, 0.97-1.07]; *P* = .50) group and the eszopiclone group (Figure 2 and eTable 8 in Supplement 1).

## Discussion

This is the first study, to our knowledge, to examine which guideline-recommended hypnotics have lower risks of monotherapy failure in treating insomnia and the risks of long-term prescription of guideline-recommended hypnotics using a large clinical dataset. Monotherapy failure occurred significantly more often for ramelteon and less often for zolpidem and triazolam compared with eszopiclone. Monotherapy discontinuation occurred significantly more often for novel hypnotics such as ramelteon and suvorexant compared with eszopiclone.

According to our results, monotherapy failure was higher with ramelteon and lower with zolpidem and triazolam when compared with eszopiclone. A recent systematic review and meta-analysis^18^ provided evidence that ramelteon is less effective than eszopiclone, with more all-cause discontinuations in indirect comparisons. Furthermore, the study concluded that eszopiclone is one of the hypnotics with the best balance between benefits and risks, whereas ramelteon has no overall material benefit.^18^ Thus, the results of the present study and previous studies on ramelteon are consistent. Notably, contrary to our hypothesis, eszopiclone, which was appraised as one of the most favorable hypnotics in a recent systematic review and meta-analysis,^18^ had more treatment failures than zolpidem and triazolam. Furthermore, a sensitivity analysis confirmed the robustness of the results, except in the case of suvorexant. The reason for this is unclear, but one possible reason is that this finding may have been influenced by differences in the rates of chronic insomnia disorders among patients prescribed guideline-recommended hypnotics. In general, Japanese psychiatrists, as sleep specialists, have the opportunity to see more patients with chronic insomnia disorders than Japanese nonpsychiatrists, and chronic insomnia disorders are less likely to improve than acute insomnia disorders.^33^ Although our study did not include a comparison between diagnoses of chronic and acute insomnia disorders or information on the department that first prescribed each hypnotic, a previous Japanese pharmacoepidemiological study^34^ reported that, among the departments that prescribed each hypnotic first, psychiatry accounted for a high percentage of prescriptions for eszopiclone (8.9%) and a low percentage of prescriptions for zolpidem (2.0%) and triazolam (1.8%), suggesting that eszopiclone may be preferred by psychiatrists. Therefore, it is possible that the eszopiclone group included more patients with chronic insomnia disorder than the zolpidem or triazolam groups, which may have resulted in more treatment failures with eszopiclone than with zolpidem or triazolam. Another possible reason for this discrepancy in results is the severity of insomnia and psychiatric symptoms. The percentage of patients with mood disorders and neurotic, stress-related, or somatoform disorders in our study was highest in the eszopiclone group and lowest in the zolpidem and triazolam groups. As eszopiclone improves not only insomnia symptoms but also core depressive and anxiety symptoms in patients with insomnia and mood or anxiety disorders,^35,36^ prescribers may have preferred eszopiclone for insomnia in patients with psychiatric disorders with high depressive or anxiety symptoms. Conversely, insufficient evidence is available to support claims on the efficacy of zolpidem when compared with eszopiclone in improving psychiatric symptoms in patients with comorbid insomnia and major depressive disorder or insomnia and anxiety disorders. The results of RCTs examining the efficacy of zolpidem against insomnia with major depressive disorder have been inconsistent; one study^37^ found zolpidem to be effective for both insomnia and depressive symptoms, while another^38^ found it to be effective against insomnia but not depressive symptoms. Additionally, zolpidem improved insomnia symptoms in patients with insomnia and anxiety disorder but did not significantly improve anxiety symptoms.^38^ These differences in evidence for eszopiclone and zolpidem for the treatment of psychiatric symptoms in comorbid psychiatric disorders may have led to eszopiclone being prescribed more often than zolpidem for insomnia with psychiatric comorbidities and possibly to it being prescribed more often for insomnia with more severe psychiatric symptoms. Insomnia symptoms have been reported to be correlated with depressive and anxiety symptoms,^39^ insomnia associated with psychiatric disorders is less likely to respond to hypnotics than insomnia without such symptoms,^32^ and patients with severe insomnia symptoms may have difficulty achieving remission with hypnotics. This study adjusted for the presence of mental disorders but not for the severity of insomnia and psychiatric symptoms. Thus, selection bias could also have contributed to more treatment failure with eszopiclone than zolpidem and triazolam in this study. Future studies with more sophisticated designs that consider diagnosis of either chronic or acute insomnia disorder and severity of insomnia, anxiety, and depression as well as physician attitudes toward prescribing hypnotics are warranted.

As per our hypothesis, this study showed more monotherapy discontinuations with suvorexant and ramelteon than with eszopiclone among patients with no monotherapy failures. Unlike BzRAs, suvorexant and ramelteon are not considered to carry a risk of dependence or rebound insomnia,^40,41,42^ hence the compelling results of this study. In addition to the pharmacologic properties of guideline-recommended hypnotics, differences in physician prescribing attitudes toward tapering of individual hypnotics may have influenced the monotherapy discontinuation. Physicians who frequently prescribed orexin receptor antagonists and melatonin receptor agonists for insomnia were reportedly more safety conscious than those who did not, physicians who frequently prescribed benzodiazepine hypnotics were less safety conscious than those who did not, and there was no difference in safety concerns between physicians who frequently prescribed nonbenzodiazepine hypnotics and those who did not.^43^ Therefore, although there are currently no known adverse events associated with long-term prescription of novel hypnotics, physicians who initially select novel hypnotics for pharmacotherapy for insomnia may be more concerned about safety than those who do not and may attempt to prescribe hypnotics on a short-term basis. However, because this study lacked information on health outcomes, adverse events, and reasons for discontinuation of hypnotics, it could not draw conclusions on whether novel hypnotics are associated with a lower risk of long-term prescription than other hypnotics. Further well-designed studies are needed to determine whether the results of this study reflect the pharmacologic characteristics of each hypnotic.

### Strengths and Limitations

This study has several strengths. First, it compared monotherapy failures among guideline-recommended hypnotics, which has not been examined previously, to our knowledge. Second, it also examined the risk of long-term prescription of hypnotics considering changes in pharmacotherapy for insomnia.

This study also has certain limitations. First, it is unclear how representative the JMDC dataset is of the general Japanese population, as it is limited to health insurance company employees and their family members younger than 75 years. Second, this study could not include certain important factors that could have affected the results, such as sociodemographic factors, adverse events, reasons for discontinuing monotherapy, and the severity of insomnia, anxiety, and depression. Although this study set monotherapy discontinuation as a surrogate measure of long-term prescription risk, monotherapy discontinuation could include cases in which the first prescribed sleep medication was discontinued due to intolerable adverse effects and no other hypnotic was prescribed thereafter. Third, this study lacked information on whether participants were referred to or offered CBT-I. However, since CBT-I is not covered by insurance and is not widely used in Japan, it is assumed that most participants did not undergo CBT-I, which would have a negligible effect in this study. Fourth, because BzRAs can lead to dependence and tolerance with long-term use, the short follow-up period in this study may have affected the results.

## Conclusions

In this cohort study, monotherapy failure was more common with ramelteon than with eszopiclone and less common with zolpidem and triazolam. Moreover, monotherapy discontinuation, set as a surrogate measure of risk of long-term use in this study, was lower with novel hypnotics such as ramelteon and suvorexant than with eszopiclone. However, these results should be interpreted with caution because several confounding factors could not be accounted for, and it is therefore unclear whether these results are due to the pharmacologic properties of the guideline-recommended hypnotics, the clinical characteristics of patients with insomnia, or the prescribing physicians’ attitudes toward the hypnotics. To determine which guideline-recommended hypnotics are most useful, RCTs directly comparing these hypnotics are needed.

## Notes

Supplement 1. **eMethods.** Comorbid Physical and Psychiatric Disorders and Cox Proportional Hazards Modeling **eFigure 1.** Specific Examples of Monotherapy Failure or Monotherapy Discontinuation Events **eFigure 2.** Assumptions of the Cox Proportional Hazards Regression Model **eFigure 3.** Participant Selection Flowchart **eFigure 4.** Cox Proportional Hazards Regression Analysis for Monotherapy Failure **eTable 1.** List of Hypnotics Covered by Insurance in Japan **eTable 2.** List of Anxiolytics Covered by Insurance in Japan **eTable 3.** List of Antidepressants Covered by Insurance in Japan **eTable 4.** List of Antipsychotics Covered by Insurance in Japan **eTable 5.** Crude and Adjusted Cox Proportional Hazards Regression Analysis **eTable 6.** Sensitivity Analyses of Cox Proportional Hazards Regression Analysis **eTable 7.** Events Occurring Within 6 Months of First Prescription of Guideline-Recommended Hypnotics Among Patients Without Failure of Guideline-Recommended Hypnotic Monotherapy **eTable 8.** Crude and Adjusted Logistic Regression Analyses

Supplement 2.
Data Sharing Statement
