# Supplementary material for: Treatment Failure and Long-Term Prescription Risk for Guideline-Recommended Hypnotics in Japan
Source: JAMA Netw Open. 2024 Apr 17;7(4):e246865. doi: 10.1001/jamanetworkopen.2024.6865 (PMC11024780; doi:10.1001/jamanetworkopen.2024.6865)
Supplement: Supplement 1. — eMethods. Comorbid Physical and Psychiatric Disorders and Cox Proportional Hazards Modeling eFigure 1. Specific Examples of Monotherapy Failure or Monotherapy Discontinuation Events eFigure 2. Assumptions of the Cox Proportional Hazards Regression Model eFigure 3. Participant Selection Flowchart eFigure 4. Cox Proportional Hazards Regression Analysis for Monotherapy Failure eTable 1. List of Hypnotics Covered by Insurance in Japan eTable 2. List of Anxiolytics Covered by Insurance in Japan eTable 3. List of Antidepressants Covered by Insurance in Japan eTable 4. List of Antipsychotics Covered by Insurance in Japan eTable 5. Crude and Adjusted Cox Proportional Hazards Regression Analysis eTable 6. Sensitivity Analyses of Cox Proportional Hazards Regression Analysis eTable 7. Events Occurring Within 6 Months of First Prescription of Guideline-Recommended Hypnotics Among Patients Without Failure of Guideline-Recommended Hypnotic Monotherapy eTable 8. Crude and Adjusted Logistic Regression Analyses [file jamanetwopen-e246865-s001.pdf]

Supplementary Online Content

Takeshima M, Yoshizawa K, Ogasawara M, et al. Treatment failure and long-term prescription risk for guideline-recommended hypnotics in Japan. *JAMA Netw Open*. 2024;7(4):e246865. doi:10.1001/jamanetworkopen.2024.6865

eMethods. Comorbid Physical and Psychiatric Disorders and Cox Proportional Hazards Modeling
eFigure 1. Specific Examples of Monotherapy Failure or Monotherapy Discontinuation Events
eFigure 2. Assumptions of the Cox Proportional Hazards Regression Model
eFigure 3. Participant Selection Flowchart
eFigure 4. Cox Proportional Hazards Regression Analysis for Monotherapy Failure
eTable 1. List of Hypnotics Covered by Insurance in Japan
eTable 2. List of Anxiolytics Covered by Insurance in Japan
eTable 3. List of Antidepressants Covered by Insurance in Japan
eTable 4. List of Antipsychotics Covered by Insurance in Japan
eTable 5. Crude and Adjusted Cox Proportional Hazards Regression Analysis
eTable 6. Sensitivity Analyses of Cox Proportional Hazards Regression Analysis
eTable 7. Events Occurring Within 6 Months of First Prescription of Guideline-Recommended Hypnotics Among Patients Without Failure of Guideline-Recommended Hypnotic Monotherapy
eTable 8. Crude and Adjusted Logistic Regression Analyses

This supplementary material has been provided by the authors to give readers additional information about their work.

© 2024 Takeshima M et al. *JAMA Network Open*.

eMethods. Comorbid Physical and Psychiatric Disorders and Cox Proportional Hazards Modeling

Comorbid physical and psychiatric disorders considered in this study based on the 10th revision of the International Statistical Classification of Diseases and Related Health Problems (ICD-10):

Psychiatric disorders

F0, mental disorders due to known physiological conditions; F1, mental and behavioral disorders due to psychoactive substance use; F2, schizophrenia, schizotypal, delusional, and other non-mood psychotic disorders; F3, mood disorders; F4, anxiety, dissociative, stress-related, somatoform and other nonpsychotic mental disorders; F5, behavioral syndromes associated with physiological disturbances and physical factors; F6, disorders of adult personality and behavior; F7, intellectual disabilities; F8, pervasive and specific developmental disorders; F9, behavioral and emotional disorders with onset usually occurring in childhood and adolescence.

Physical disorders

Hypertension, hyperlipidemia, diabetes mellitus, cancer, rheumatoid arthritis, Parkinson's disease, multiple sclerosis, bronchial asthma, chronic obstructive pulmonary disease, coronary artery disease, pulmonary embolism, deep vein thrombosis, hypothyroidism, subarachnoid hemorrhage, nontraumatic intracerebral hemorrhage, cerebral infarction, heart failure, and inflammatory bowel disease.

Testing the Cox proportional hazards modeling assumptions:

The Cox proportional hazards model assumptions were tested using the STATA procedure 'estat phtest' (STATA 13.0; Stata Corp, College Station, TX) and by plotting Schoenfeld residuals against time-period of the first prescription of hypnotics. There was no evidence of deviation from proportional hazards for time-period ($p > 0.05$, with no apparent slope in the residual plots) (eFigure 1). Therefore, a proportional hazard was established.

© 2024 Takeshima M et al. *JAMA Network Open*.

eFigure 1. Specific Examples of Monotherapy Failure or Monotherapy Discontinuation Events

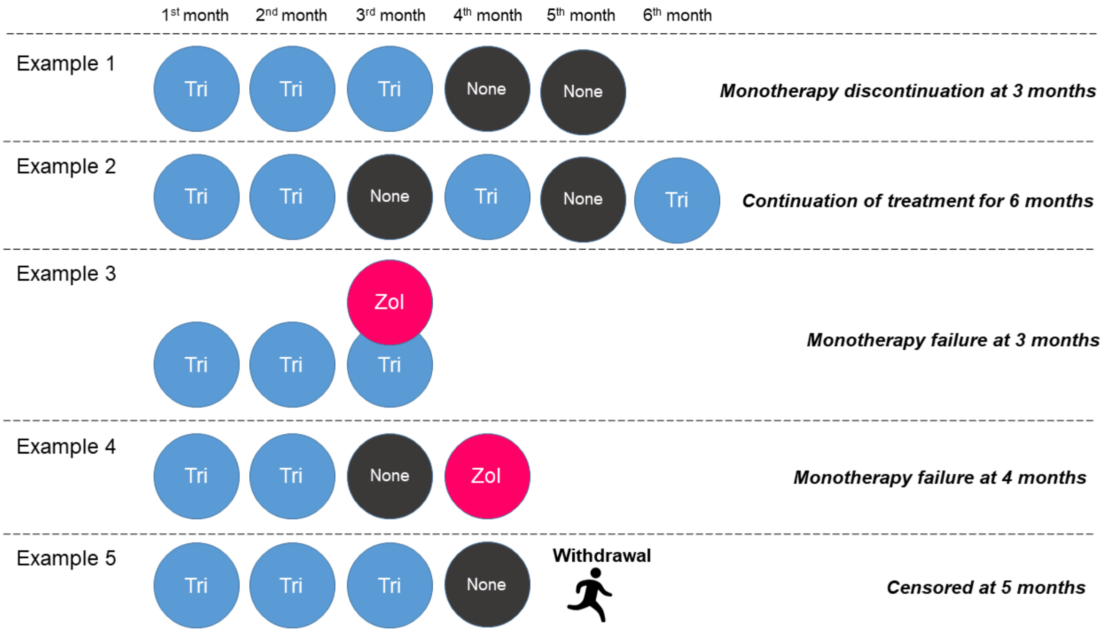

Abbreviations: Tri, triazolam; Zol, zolpidem

© 2024 Takeshima M et al. *JAMA Network Open*.

eFigure 2. Assumptions of the Cox Proportional Hazards Regression Model

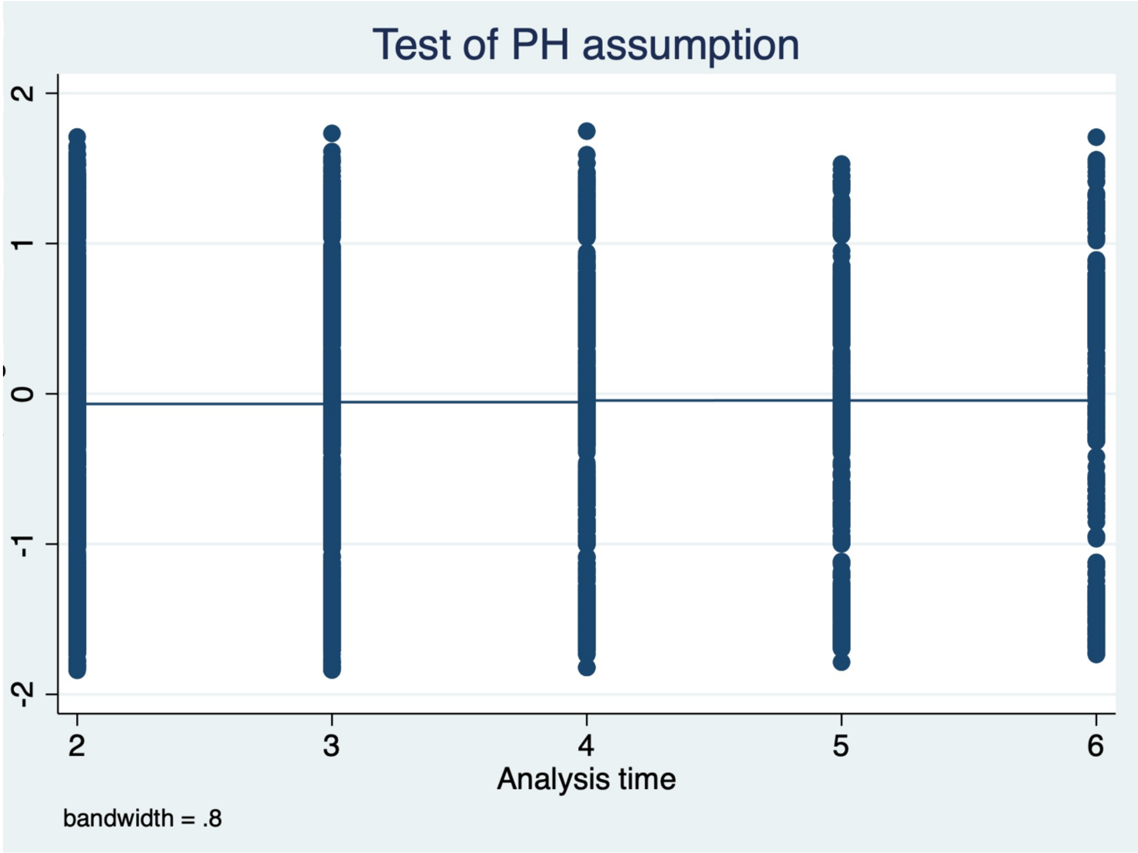

© 2024 Takeshima M et al. *JAMA Network Open*.

eFigure 3. Participant Selection Flowchart

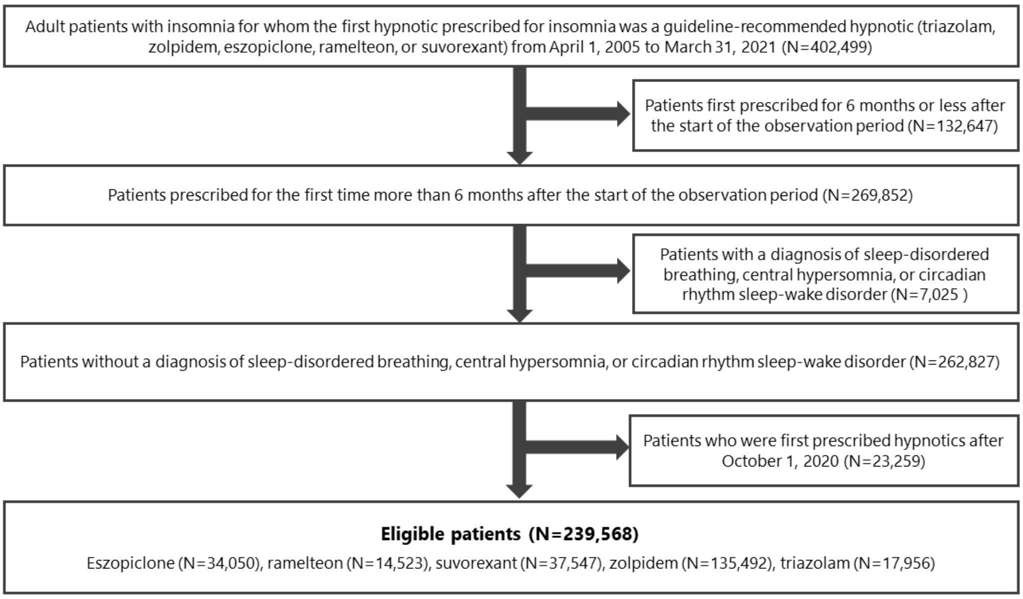

© 2024 Takeshima M et al. *JAMA Network Open*.

eFigure 4. Cox Proportional Hazards Regression Analysis for Monotherapy Failure

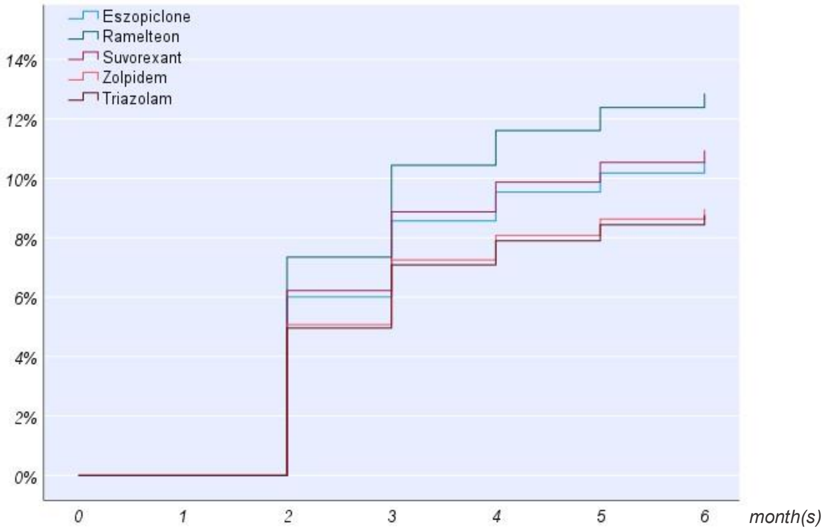

© 2024 Takeshima M et al. *JAMA Network Open*.

eTable 1. List of Hypnotics Covered by Insurance in Japan

Generic name	ATC code	Introduction
**Benzodiazepine**		
Brotizolam	N05CD09	Prior to 2005
Estazolam	N05CD04	Prior to 2005
Etizolam	N05BA19	Prior to 2005
Flunitrazepam	N05CD03	Prior to 2005
Flurazepam	N05CD01	Prior to 2005
Haloxazolam	–	Prior to 2005
Lormetazepam	N05CD06	Prior to 2005
Nimetazepam	N05CD15	Prior to 2005
Nitrazepam	N05CD02	Prior to 2005
Quazepam	N05CD10	Prior to 2005
Rilmazafone	–	Prior to 2005
Triazolam	N05CD05	Prior to 2005
**Non-benzodiazepine**		
Eszopiclone	N05CF04	April 2012
Zolpidem	N05CF02	Prior to 2005
Zopiclone	N05CF01	Prior to 2005
**Melatonin receptor agonist**		
Melatonin[A]	N05CH01	June 2020
Ramelteon	N05CH02	July 2010
**Orexin receptor antagonist**		
Lemborexant	–	June 2020
Suvorexant	N05CM19	November 2014

Note: [A]Insurance coverage is available for difficulty falling asleep associated with childhood neurodevelopmental disorders.

Abbreviation: ATC, Anatomical Therapeutic Chemical classification 2021

© 2024 Takeshima M et al. *JAMA Network Open*.

eTable 2. List of Anxiolytics Covered by Insurance in Japan

Generic name	ATC code
Alprazolam	N05BA12
Bromazepam	N05BA08
Chlordiazepoxide	N05BA02
Clorazepate	N05BA05
Clotiazepam	N05BA21
Cloxazolam	N05BA22
Diazepam	N05BA01
Etizolam	N05BA19
Fludiazepam	N05BA17
Flutazolam	–
Flutoprazepam	–
Hydroxyzine	N05BB01
Loflazepate	N05BA18
Lorazepam	N05BA06
Medazepam	N05BA03
Mexazolam	N05BA25
Oxazepam	N05BA04
Oxazolam	–
Prazepam	N05BA11
Tandospirone	–
Tofisopam	N05BA23

ATC, Anatomical Therapeutic Chemical classification 2021

© 2024 Takeshima M et al. *JAMA Network Open*.

eTable 3. List of Antidepressants Covered by Insurance in Japan

Generic name	ATC code
Amitriptyline	N06AA09
Amoxapine	N06AA17
Clomipramine	N06AA04
Desipramine	N06AA01
Dosulepin	N06AA16
Duloxetine	N06AX21
Escitalopram	N06AB10
Fluvoxamine	N06AB08
Imipramine	N06AA02
Lofepramine	N06AA07
Maprotiline	N06AA21
Mianserin	N06AX03
Milnacipran	N06AX17
Mirtazapine	N06AX11
Nortriptyline	N06AA10
Paroxetine	N06AB05
Safrazine	–
Sertraline	N06AB06
Setiptiline	–
Sulpiride	N05AL01
Trazodone	N06AX05
Trimipramine	N06AA06
Venlafaxine	N06AX16
Vortioxetine	N06AX26

ATC, Anatomical Therapeutic Chemical classification 2021

© 2024 Takeshima M et al. *JAMA Network Open*.

eTable 4. List of Antipsychotics Covered by Insurance in Japan

Generic name	ATC code
Aripiprazole	N05AX12
Asenapine	N05AH05
Blonanserin	–
Blonanserin (tape)	–
Brexpiprazole	N05AX16
Bromperidol	N05AD06
Carpipramine	–
Chlorpromazine	N05AA01
Clocapramine	–
Clozapine	N05AH02
Floropipamide	–
Fluphenazine	N05AB02
Haloperidol	N05AD01
Levomepromazine	N05AA02
Lurasidone	N05AE05
Moperone	N05AD04
Mosapramine	N05AX10
Nemonapride	–
Olanzapine	N05AH03
Oxypertine	N05AE01
Paliperidone	N05AX13
Perospirone	–
Perphenazine	N05AB03
Pimozide	N05AG02
Prochlorperazine	N05AB04
Propericyazine	–
Quetiapine	N05AH04
Risperidone	N05AX08
Spiperone	–
Sulpiride	N05AL01
Sultopride	N05AL02
Tiapride	N05AL03
Thioridazine	N05AC02
Timiperone†	–
Trifluoperazine	N05AB06
Zotepine	N05AX11

ATC, Anatomical Therapeutic Chemical classification 2021

© 2024 Takeshima M et al. *JAMA Network Open*.

eTable 5. Crude and Adjusted Cox Proportional Hazards Regression Analysis

	cHR (95% CI)[a]	P-value	aHR (95% CI)[a,b]	P-value
Guideline-recommended hypnotic				
Eszopiclone	1 [Reference]		1 [Reference]	
Ramelteon	1.28 (1.22–1.35)	<.001[d]	1.23 (1.17–1.30)	<.001[d]
Suvorexant	1.07 (1.03–1.12)	.002[c]	1.04 (0.99–1.08)	.091
Zolpidem	0.74 (0.71–0.76)	<.001[d]	0.84 (0.81–0.87)	<.001[d]
Triazolam	0.78 (0.74–0.83)	<.001[d]	0.82 (0.78–0.87)	<.001[d]
Age group				
20–39 years	1 [Reference]		1 [Reference]	
40–64 years	0.75 (0.73–0.77)	<.001[d]	0.88 (0.86–0.91)	<.001[d]
65 years or more	0.64 (0.60–0.68)	<.001[d]	0.85 (0.80–0.90)	<.001[d]
Sex				
Male	1 [Reference]		1 [Reference]	
Female	0.84 (0.82–0.86)	<.001[d]	0.90 (0.88–0.93)	<.001[d]
Prescription instruction				
As-needed only	1 [Reference]		1 [Reference]	
Regular	1.64 (1.59–1.69)	<.001[d]	1.45 (1.41–1.50)	<.001[d]
Physical comorbidities				
Hypertension	0.84 (0.81–0.87)	<.001[d]	1.02 (0.98–1.06)	.36
Hyperlipidemia	0.86 (0.83–0.89)	<.001[d]	0.99 (0.95–1.04)	.73
Diabetes	0.88 (0.85–0.92)	<.001[d]	1.03 (0.98–1.08)	.32
Cancer	0.93 (0.88–0.97)	.002[c]	1.37 (1.30–1.44)	<.001[d]
Rheumatoid arthritis	0.90 (0.79–1.02)	.10	1.08 (0.95–1.23)	.24
Parkinson's disease	1.39 (1.19–1.62)	<.001[d]	1.09 (0.93–1.28)	.28
Multiple sclerosis	0.89 (0.57–1.38)	.59	1.16 (0.75–1.80)	.50
Asthma	0.91 (0.86–0.96)	<.001[d]	1.01 (0.96–1.07)	.61
Chronic obstructive pulmonary disease	1.08 (0.92–1.26)	.34	1.20 (1.02–1.41)	.03[c]
Coronary artery disease	0.89 (0.83–0.95)	<.001[d]	1.01 (0.94–1.09)	.82
Pulmonary embolism	1.06 (0.76–1.47)	.74	1.19 (0.85–1.66)	.32
Deep vein thrombosis	0.82 (0.70–0.96)	.02[c]	0.97 (0.82–1.14)	.67
Hypothyroid	1.09 (0.98–1.21)	.12	1.10 (0.99–1.22)	.08
Subarachnoid hemorrhage	1.04 (0.80–1.34)	.78	1.31 (1.01–1.70)	.04[c]
Nontraumatic intracerebral hemorrhage	1.06 (0.86–1.31)	.57	1.30 (1.06–1.61)	.01[c]

© 2024 Takeshima M et al. *JAMA Network Open*.

Cerebral infarction	1.01 (0.91–1.13)	.82	1.20 (1.07–1.34)	.001*
Heart failure	1.00 (0.93–1.07)	1.00	1.19 (1.10–1.28)	<.001[d]
Inflammatory bowel disease	1.04 (0.88–1.23)	.69	1.23 (1.04–1.46)	.02[c]
Psychiatric comorbidities				
Organic mental disorder	1.18 (0.99–1.40)	.07	1.01 (0.84–1.20)	.96
Substance use disorder	1.34 (1.18–1.53)	<.001[d]	1.23 (1.08–1.41)	.002[c]
Schizophrenia	1.64 (1.55–1.74)	<.001[d]	1.00 (0.93–1.08)	.95
Mood disorders	2.21 (2.15–2.26)	<.001[d]	1.47 (1.41–1.53)	<.001[d]
Anxiety disorder	1.76 (1.71–1.80)	<.001[d]	1.28 (1.25–1.32)	<.001[d]
Behavioral and emotional disorders	1.59 (1.46–1.72)	<.001[d]	1.20 (1.10–1.30)	<.001[d]
Personality disorder	1.88 (1.50–2.35)	<.001[d]	1.26 (1.00–1.57)	.046[c]
Intellectual disability	1.12 (0.85–1.48)	.43	0.88 (0.66–1.16)	.36
Psychological development disorder	1.35 (1.19–1.54)	<.001[d]	0.87 (0.75–1.00)	.04[c]
BEDCA and UMD	1.74 (1.58–1.93)	<.001[d]	1.25 (1.13–1.39)	<.001**
Psychotropic medications				
Antidepressants	2.14 (2.09–2.20)	<.001[d]	1.24 (1.19–1.30)	<.001[d]
Anxiolytics	1.82 (1.77–1.87)	<.001[d]	1.25 (1.21–1.29)	.001[c]
Antipsychotics	1.66 (1.59–1.74)	<.001[d]	1.24 (1.16–1.32)	<.001[d]

Note:

[a] Large hazard ratios indicate more monotherapy failures, whereas small hazard ratios indicate fewer monotherapy failures.

[b] Adjusted for age group (20–39, 40–64, ≥65 years), sex, administration instructions of the first prescribed hypnotic (as-needed only or regular prescription), concomitant anxiolytics, concomitant antidepressants, concomitant antipsychotics, comorbid psychiatric disorders, and comorbid physical disorders in the month of the first prescription.

[c] P-value <.05

[d] P-value <.001

Abbreviations: aHR, adjusted hazard ratio; BEDCA, behavioral and emotional disorders with onset usually occurring in childhood and adolescence; CI, confidence interval; COPD, chronic obstructive pulmonary disease; cHR, crude hazard ratio; UMD, unspecified mental disorder

© 2024 Takeshima M et al. *JAMA Network Open*.

eTable 6. Sensitivity Analyses of Cox Proportional Hazards Regression Analysis

	Adjusted HR (95%CI)[a,b]	P-value
Under 64 years old[c]		
Eszopiclone	1 [Reference]	
Ramelteon	1.21 (1.15–1.28)	<.001[k]
Suvorexant	1.04 (0.99–1.08)	.12
Zolpidem	0.84 (0.80–0.87)	<.001[k]
Triazolam	0.82 (0.77–0.87)	<.001[k]
Without mood disorder[d]		
Eszopiclone	1 [Reference]	
Ramelteon	1.24 (1.15–1.33)	<.001[k]
Suvorexant	1.05 (0.99–1.11)	.12
Zolpidem	0.80 (0.77–0.84)	<.001[k]
Triazolam	0.77 (0.71–0.83)	<.001[k]
Without anxiety disorder[e]		
Eszopiclone	1 [Reference]	
Ramelteon	1.28 (1.20–1.37)	<.001[k]
Suvorexant	1.06 (1.01–1.12)	.03[j]
Zolpidem	0.84 (0.80–0.88)	<.001[k]
Triazolam	0.83 (0.78–0.90)	<.001[k]
Without mood or anxiety disorder[f]		
Eszopiclone	1 [Reference]	
Ramelteon	1.34 (1.23–1.47)	<.001[k]
Suvorexant	1.09 (1.01–1.17)	.02[j]
Zolpidem	0.80 (0.75–0.85)	<.001[k]
Triazolam	0.77 (0.70–0.85)	<.001[k]
Without any psychiatric disorder[g]		
Eszopiclone	1 [Reference]	
Ramelteon	1.32 (1.20–1.45)	<.001[k]
Suvorexant	1.10 (1.02–1.18)	.02[j]
Zolpidem	0.79 (0.75–0.84)	<.001[k]
Triazolam	0.75 (0.68–0.83)	<.001[k]
Monotherapy failure considering trazodone[h]		
Eszopiclone	1 [Reference]	
Ramelteon	1.23 (1.17–1.30)	<.001[k]
Suvorexant	1.06 (1.02–1.10)	.008[j]
Zolpidem	0.84 (0.81–0.87)	<.001[k]
Triazolam	0.81 (0.76–0.85)	<.001[k]

© 2024 Takeshima M et al. *JAMA Network Open*.

After November 2014 [i]		
Eszopiclone	1 [Reference]	
Ramelteon	1.24 (1.18–1.31)	<.001[k]
Suvorexant	1.04 (0.99–1.08)	.10
Zolpidem	0.83 (0.80–0.87)	<.001[k]
Triazolam	0.80 (0.75–0.85)	<.001[k]

Note:

[a] Large hazard ratios indicate more monotherapy failures, whereas small hazard ratios indicate fewer monotherapy failures.

[b] Adjusted for age group (20–39, 40–64, ≥65 years), sex, administration instructions of the first prescribed hypnotic (as-needed only or regular prescription), concomitant anxiolytics, concomitant antidepressants, concomitant antipsychotics, comorbid psychiatric disorders, and comorbid physical disorders in the month of the first prescription.

[c] Sensitivity analysis in patients under 64 years of age prescribed guideline-recommended sleep medications for the first time.

[d] Sensitivity analysis in patients without mood disorder

[e] Sensitivity analysis in patients without anxiety disorder

[f] Sensitivity analysis in patients without mood or anxiety disorder

[g] Sensitivity analysis in patients without any psychiatric disorder

[h] Sensitivity analysis of monotherapy failure considering trazodone

[i] Sensitivity analysis of patients who were first prescribed hypnotics after November 2014, when all guideline-recommended hypnotics became available for prescription in Japan.

[j] P-value <.05

[k] P-value <.001

Abbreviations: CI, confidence interval; HR, hazard ratio

© 2024 Takeshima M et al. *JAMA Network Open*.

eTable 7. Events Occurring Within 6 Months of First Prescription of Guideline-Recommended Hypnotics Among Patients Without Failure of Guideline-Recommended Hypnotic Monotherapy

	Eszopiclone N=29,988	Ramelteon N=12,330	Suvorexant N=32,780	Zolpidem N=123,426	Triazolam N=16,266	Total N=214,790
Monotherapy discontinuation	24,857 (82.9%)	10,468 (84.9%)	27,538 (84.0%)	105,138 (85.2%)	13,622 (83.7%)	181,623 (84.6%)
Continuation for 6 months	3,627 (12.1%)	1,268 (10.3%)	3,564 (10.9%)	12,915 (10.5%)	1,897 (11.7%)	23,271 (10.8%)
Censor	1,504 (5.0%)	594 (4.8%)	1,678 (5.1%)	5,373 (4.4%)	747 (4.6%)	9,896 (4.6%)

© 2024 Takeshima M et al. *JAMA Network Open*.

eTable 8. Crude and Adjusted Logistic Regression Analyses

	cOR (95% CI)[a]	P-value	aOR (95% CI)[a,b]	P-value
Guideline-recommended hypnotic				
Eszopiclone	1 [Reference]		1 [Reference]	
Ramelteon	1.16 (1.10–1.23)	<.001[d]	1.31 (1.24–1.40)	<.001[d]
Suvorexant	1.08 (1.04–1.13)	<.001[d]	1.20 (1.15–1.26)	<.001[d]
Zolpidem	1.19 (1.15–1.23)	<.001[d]	1.00 (0.97–1.04)	.97
Triazolam	1.06 (1.01–1.12)	.02[c]	1.02 (0.97–1.07)	.50
Age group				
20–39 years	1 [Reference]		1 [Reference]	
40–64 years	1.08 (1.06–1.11)	<.001[d]	0.92 (0.90–0.95)	<.001[d]
65 years or more	0.84 (0.80–0.87)	<.001[d]	0.65 (0.62–0.69)	<.001[d]
Sex				
Male	1 [Reference]		1 [Reference]	
Female	1.19 (1.16–1.22)	<.001[d]	1.09 (1.06–1.11)	<.001[d]
Prescription instruction				
As-needed only	1 [Reference]		1 [Reference]	
Regular	0.48 (0.47–0.50)	<.001[d]	0.51 (0.49–0.52)	<.001[d]
Physical comorbidities				
Hypertension	0.79 (0.77–0.82)	<.001[d]	0.70 (0.68–0.73)	<.001[d]
Hyperlipidemia	0.84 (0.81–0.86)	<.001[d]	0.88 (0.84–0.91)	<.001[d]
Diabetes	0.83 (0.80–0.86)	<.001[d]	0.86 (0.83–0.90)	<.001[d]
Cancer	1.18 (1.13–1.24)	<.001[d]	0.86 (0.82–0.90)	<.001[d]
Rheumatoid arthritis	0.92 (0.82–1.03)	.14	0.84 (0.75–0.95)	.003[c]
Parkinson's disease	0.43 (0.37–0.49)	<.001[d]	0.63 (0.54–0.73)	<.001[d]
Multiple sclerosis	1.10 (0.74–1.65)	.63	0.79 (0.52–1.19)	.25
Asthma	1.03 (0.98–1.08)	.23	0.99 (0.94–1.05)	.81
Chronic obstructive pulmonary disease	0.99 (0.85–1.15)	.86	1.00 (0.85–1.17)	.99
Coronary artery disease	1.08 (1.01–1.15)	.02[c]	1.23 (1.15–1.32)	<.001[d]
Pulmonary embolism	0.98 (0.72–1.33)	.88	0.76 (0.55–1.05)	.09
Deep vein thrombosis	1.44 (1.24–1.68)	<.001[d]	1.27 (1.08–1.49)	.004[c]
Hypothyroid	0.89 (0.81–0.98)	.02[c]	0.96 (0.87–1.07)	.48
Subarachnoid hemorrhage	2.15 (1.54–3.00)	<.001[d]	2.01 (1.43–2.82)	<.001[d]
Nontraumatic intracerebral hemorrhage	1.23 (0.99–1.53)	.06	1.25 (1.00–1.56)	.06
Cerebral infarction	0.87 (0.79–0.96)	.004[c]	0.94 (0.84–1.04)	.20

© 2024 Takeshima M et al. *JAMA Network Open*.

Heart failure	1.01 (0.94–1.08)	.87	1.00 (0.93–1.08)	.92
Inflammatory bowel disease	1.02 (0.87–1.20)	.80	0.79 (0.67–0.94)	.006[c]
Psychiatric comorbidities				
Organic mental disorder	0.64 (0.55–0.75)	<.001[d]	0.76 (0.65–0.90)	.001[c]
Substance use disorder	0.81 (0.71–0.92)	.002[c]	0.90 (0.78–1.03)	.12
Schizophrenia	0.44 (0.42–0.47)	<.001[d]	0.80 (0.75–0.87)	<.001[d]
Mood disorders	0.40 (0.39–0.41)	<.001[d]	0.60 (0.58–0.63)	<.001[d]
Anxiety disorder	0.57 (0.55–0.58)	<.001[d]	0.76 (0.73–0.78)	<.001[d]
Behavioral and emotional disorders	0.64 (0.59–0.70)	<.001[d]	0.88 (0.81–0.97)	.006[c]
Personality disorder	0.57 (0.45–0.74)	<.001[d]	0.89 (0.69–1.15)	.38
Intellectual disability	0.49 (0.39–0.61)	<.001[d]	0.59 (0.47–0.76)	<.001[d]
Psychological development disorder	0.48 (0.43–0.54)	<.001[d]	0.70 (0.62–0.80)	<.001[d]
BEDCA and UMD	0.49 (0.44–0.55)	<.001[d]	0.68 (0.61–0.77)	<.001[d]
Psychotropic medications				
Antidepressants	0.39 (0.38–0.40)	<.001[d]	0.67 (0.64–0.69)	<.001[d]
Anxiolytics	0.48 (0.45–0.50)	<.001[d]	0.82 (0.79–0.84)	<.001[d]
Antipsychotics	0.52 (0.51–0.53)	<.001[d]	0.67 (0.63–0.72)	<.001[d]

Note:

[a] Large odds ratios indicate more successful discontinuations, whereas small odds ratios indicate fewer successful discontinuations.

[b] Adjusted for age group (20–39, 40–64, ≥65 years), sex, administration instructions of the first prescribed hypnotic (as-needed only or regular prescription), concomitant anxiolytics, concomitant antidepressants, concomitant antipsychotics, comorbid psychiatric disorders, and comorbid physical disorders in the month of the first prescription.

[c] P-value <.05

[d] P-value <.001

Abbreviations: aOR, adjusted odds ratio; BEDCA, behavioral and emotional disorders with onset usually occurring in childhood and adolescence; CI, confidence interval; cOR, crude odds ratio; UMD, unspecified mental disorder

© 2024 Takeshima M et al. *JAMA Network Open*.
