# Supplementary material for: Treatment Failure and Long-Term Prescription Risk for Guideline-Recommended Hypnotics in Japan
Source: JAMA Netw Open. 2024 Apr 17;7(4):e246865. doi: 10.1001/jamanetworkopen.2024.6865 (PMC11024780; doi:10.1001/jamanetworkopen.2024.6865)
Supplement: Supplement 2. — Data Sharing Statement [file jamanetwopen-e246865-s002.pdf]

# Data Sharing Statement

Takeshima. Treatment Failure and Long-Term Prescription Risk for Guideline-Recommended Hypnotics in Japan. *JAMA Netw Open*. Published April 17, 2024.
doi:10.1001/jamanetworkopen.2024.6865

**Data**
**Data available:** No

**Additional Information**
**Explanation for why data not available:** The data supporting the results of this study are available from JMDC, but the availability of these data is restricted. The data were used under license for the current study and are not publicly available. However, the data are available from the authors upon reasonable request and with permission from JMDC.
